# Told by a Snake Charmer

**Published:** 1889-10

**Authors:** 


### TOLD BY A SNAKE CHARMER.

### HE HAS BEEN BITTEN FORTY-NINE TIMES—HIS REMEDY FOR SNAKE BITE.

"I have been bitten forty-nine times by rattlesnakes," said C. J. Ironmonger, of this city, to a *Republican* reporter yesterday:

"Yes, sir, forty-nine times; either while catching them, handling them, or performing before the public with them. I am known as the great serpent charmer or tamer. I have handled thousands of venomous serpents, and twenty-three years ago I performed in the Bella Union Theater as a snake charmer, when it was managed by Mrs. Tellow and son.

"I have often heard that if a rattlesnake bite a person, the sore, even if healed, will break out again every twelve months. That is not so. If you are once cured you are cured forever. I have often read in the newspapers of snakes charming little children, and of children feeding them without being bitten. That is all nonsense.

"No venomous snake will eat dead food; it must be live food that he catches himself, and he will not take food while caged up, not even the most inviting morsel. They can live a remarkably long time without food. I once saw a rattlesnake in Marysville, in a cage, that had not taken food for eleven years. I have kept many of them from five to eleven years without food. They will, however, drink water, I suppose, to keep their poison replenished.

"Rattlers are epicureans. They will crawl out of their dens in the spring, and if they catch a quail, a young rabbit, or a squirrel during the summer, they are satisfied. If not, they will take their drink of water in the fall and go to their dens and wait until the next spring for their breakfast."

"What remedies do you use for snake bites?" was asked.

"I put an ounce of ammonia into a two-ounce vial. Then I add a dozen or so leaves of the mistletoe. The leaves soon dissolve, and the liquid becomes of a reddish color. Put eighteen or twenty drops of this liquid into a tumbler half full of water, and drink it as soon as you can after being bitten. Then drink a pint of whiskey. After that you must wait fifteen or twenty minutes, and if you feel no signs of inebriation repeat the dose ; but the moment you feel the effects of the whiskey drink no more, but you may take another dose of the ammoniated liquid.

"Some people when bitten by a snake keep pouring down whiskey until they get thoroughly drunk, but in such cases the remedy is worse than the disease. Those who know me say that I am poison proof, but that is not true, while at the same time a snake bite that would kill some men would injure me very little Harmless snakes are all gourmands and want to be swallowing all the time."

"Do you believe that snakes can fascinate or charm other animals ?" asked the reporter.

"That is an error," was the reply. "No serpent has that faculty. Every animal has its own instinctive way of taking its prey—by stealth, agility, brute force, or strength. My experience during the last forty years confirms my belief. I have often heard birds making a great noise in a swamp, and I used to say, 'There's a snake charming a bird.' I would go down there to see what was going on, and sure enough there would be a black snake going along slowly from one branch of a tree to another. There would be a catbird or some other kind of a bird flying around the snake, greatly excited, while the snake moved slowly toward a nest to rob it of its young.

"The maternal instinct of a mother bird makes her forget the danger in a great measure. So she flies around and over the snake and flutters along just ahead of him, trying to lead him in an opposite direction, but all to no purpose, and as he comes near the nest she becomes more desperate.

"She pecks at his eyes, beats him with her wings, and like a flash he grabs her, for he would as soon have her as the young, because she is a larger mouthful if not so delicious.

"She was not spell-bound ; she had all the use of her muscles and could leave him at her will.

"At the Sacramento State Fair some twenty odd years ago I was bitten by a rattler in the bowels, and he left his fangs behind. I performed in the tent until nine o'clock at night. In closing up I felt around my person and pulled all of the snakes out of my bosom, as I thought, but I overlooked one. I went around the town to see the sights, playing a game or two, and started for my hotel.

"It was called the Oro then, but the name is changed now. Then I threw myself across the bed, and the first thing I knew I was bitten. I was smothering him. I grabbed him by the head through my shirt, and gave him a twist. Hence I broke his fangs. I held him up to the moonlight and found him to be a favorite of mine.

"I had no remedies with me, and the drug stores were shut up at that hour. At last I found a doctor named Logan, who gave me a bottle of whiskey and twenty-five drops of ammonia, and I was soon all right, but the fangs remained in me. I began to grow sick at last, and the wound had to be opened afresh and the fangs extracted. It was three months after I was bitten that the network of fat that covers the bowels began to inflame, and it came near killing me.

"I have not said anything about tarantulas, scorpions and centipedes, but they are all in my line. I have been stung by all of them, and I use the same remedy."—*Fresno Republican.*

